# Clinical and Economic Trade-Offs in Antifungal Therapy for Invasive Aspergillosis and Mucormycosis: A Markov Model–Based Analysis

**DOI:** 10.3390/jof12080608

**Published:** 2026-08-14

**Authors:** Alejandro Rico Mendoza, Alexandra Porras Ramirez, Liliana Encinales, Oscar Madiedo, Marianna Carrillo Encinales, Juan Pablo Duque Bolivar, Juan Fernando Ramon Cuellar, Jorge Andrés Urquijo Mendez, Zuly Moreno Perilla, Roberto Jurado Zambrano

**Affiliations:** 1 Grupo de Medicina Comunitaria y Salud Colectiva, Universidad El Bosque, Bogotá 110121, Colombia; ricoalejandro@unbosque.edu.co (A.R.M.); jurquijom@unbosque.edu.co (J.A.U.M.); zulym88@hotmail.com (Z.M.P.); 2 Allied Research Society Colombia SAS, Barranquilla 080001, Colombia; liliana_encinales@yahoo.com (L.E.); icioeml@gmail.com (O.M.); marianna.carrillo2004@gmail.com (M.C.E.); juanduque31@gmail.com (J.P.D.B.); 3 Grupo de Neurocirugía, Fundación Santa Fe de Bogotá, Bogotá 110111, Colombia; juanfernandoramon@yahoo.com; 4 Departamento de Medicina Interna, Los Cobos Medical Center, Bogotá 110121, Colombia; jrjuradoz@loscobosmc.com

**Keywords:** cost-effectiveness, invasive aspergillosis, mucormycosis, antifungal therapy, Markov model, isavuconazole, voriconazole, liposomal amphotericin B, Colombia, health economics

## Abstract

Invasive aspergillosis and mucormycosis are life-threatening fungal infections characterized by high early mortality, substantial toxicity, and intensive healthcare resource utilization. While multiple antifungal therapies are recommended in international guidelines, their comparative clinical and economic value remains uncertain, particularly in resource-limited settings. We developed a hybrid decision tree and Markov model to evaluate antifungal strategies, including liposomal amphotericin B, isavuconazole, voriconazole, posaconazole, and caspofungin, from the perspective of the Colombian healthcare system over a 6-month horizon. Health states included clinical response, treatment failure, toxicity, and death. Outcomes included quality-adjusted life-years (QALYs), costs, incremental cost-effectiveness ratios (ICERs), and net monetary benefit (NMB). Deterministic and probabilistic sensitivity analyses were performed. In invasive aspergillosis, isavuconazole provided a modest incremental benefit over voriconazole (+0.022 QALYs), driven primarily by improved tolerability, at an incremental cost of COP 3.4 million, yielding an ICER of COP 154.5 million/QALY. In mucormycosis, liposomal amphotericin B yielded greater survival benefits (+0.035 QALYs) but at substantially higher costs (ICER: COP 494.3 million/QALY). Across willingness-to-pay thresholds, isavuconazole and voriconazole demonstrated more favorable economic profiles, owing to lower toxicity and reduced hospitalizations. These findings highlight that the clinical–economic value of antifungal therapy is primarily determined by early survival gains, toxicity burden, and front-loaded costs. Strategies minimizing toxicity and hospitalization appear more efficient in constrained health systems, whereas survival-maximizing approaches may be justified in severe disease. This integrated analysis supports context-specific antifungal decision-making in the management of invasive fungal infections.

## 1. Introduction

Invasive fungal infections (IFIs) remain a major cause of morbidity, mortality, and healthcare expenditure among immunocompromised patients worldwide. Among these infections, invasive aspergillosis (IA) and mucormycosis are particularly important because of their high case-fatality rates, diagnostic challenges, and substantial resource utilization. Despite advances in antifungal therapy and supportive care, mortality rates for IA frequently exceed 30%. In contrast, mucormycosis is associated with mortality rates ranging from 40% to 80%, depending on the underlying condition, anatomical site of infection, and timing of treatment initiation [1,2].

Invasive aspergillosis is predominantly caused by Aspergillus fumigatus. It occurs mainly in patients with hematologic malignancies, prolonged neutropenia, hematopoietic stem cell transplantation, solid organ transplantation, and other forms of severe immunosuppression [1]. Mucormycosis, although less frequent, is a rapidly progressive opportunistic infection caused by fungi of the order Mucorales and is particularly associated with uncontrolled diabetes mellitus, hematological malignancies, corticosteroid exposure, and transplantation [2]. Both diseases require prompt diagnosis and initiation of antifungal therapy because delays in treatment are associated with significantly worse clinical outcomes [2,3].

In Colombia, invasive fungal infections represent an increasingly important public health challenge due to the growing number of patients living with cancer, diabetes mellitus, transplantation, autoimmune diseases, and other conditions requiring immunosuppressive therapy. Invasive aspergillosis remains one of the most frequently reported mold infections among high-risk populations, particularly in hematology and intensive care settings [4]. Although mucormycosis is less common, it is associated with substantial mortality, prolonged hospitalization, and high treatment costs, especially among patients with uncontrolled diabetes and severe immunosuppression [2]. Despite their clinical relevance, epidemiological and economic data on these infections remain limited in Colombia, creating uncertainty about the optimal allocation of healthcare resources and antifungal treatment strategies.

Several antifungal agents are currently available for the management of IA and mucormycosis, including liposomal amphotericin B, voriconazole, isavuconazole, posaconazole, itraconazole, and echinocandins, with the latter used in selected clinical scenarios [1,2]. While randomized clinical trials have demonstrated the efficacy of modern triazoles and amphotericin-based regimens, treatment decisions frequently require balancing clinical effectiveness, toxicity profiles, drug–drug interactions, hospitalization requirements, and economic consequences [3,5]. In resource-constrained healthcare systems, these considerations are particularly relevant, as antifungal therapies account for a substantial share of total healthcare expenditures.

Several methodological approaches have been used to evaluate the clinical and economic consequences of antifungal therapies, including cost-minimization analyses, budget impact models, decision-tree models, and state-transition models. Decision trees are useful for evaluating short-term outcomes but have limited ability to capture recurrent events, treatment switching, toxicity-related transitions, and changes in disease status over time. In contrast, Markov models allow patients to transition between clinically relevant health states throughout the course of the disease. They are particularly suitable for conditions characterized by dynamic progression, recurrent complications, and substantial mortality risk [6,7]. Because IA and mucormycosis involve changes in treatment response, toxicity, hospitalization, and survival over time, a hybrid decision tree–Markov framework was considered the most appropriate methodological approach for evaluating the clinical and economic trade-offs associated with antifungal therapy.

Although several economic evaluations have assessed antifungal therapies in North America and Europe, evidence from Latin America remains scarce, and results from high-income countries may not be directly transferable to middle-income healthcare systems because of differences in drug acquisition costs, hospitalization expenses, healthcare resource utilization, and treatment accessibility [8,9]. Consequently, locally informed economic analyses are required to support evidence-based decision-making and efficient resource allocation.

The objective of this study was to evaluate the clinical outcomes, quality-adjusted life-years (QALYs), costs, incremental cost-effectiveness ratios (ICERs), and net monetary benefits (NMBs) associated with alternative antifungal treatment strategies for invasive aspergillosis and mucormycosis from the perspective of the Colombian healthcare system using a hybrid decision tree–Markov model.

## 2. Methods

### 2.1. Study Design and Theoretical Framework

Using a decision-analytic framework, we performed a cost-utility analysis to evaluate antifungal treatment strategies for invasive aspergillosis and mucormycosis. The model integrated an initial decision tree to capture early clinical activities, along with a state-transition (Markov) model for the disease’s time evolution. The hybrid structure was chosen to capture the clinical course of invasive fungal infections, in which acute-onset, early-onset, and high mortality, along with temporal variability, characterize the disease stage. The analyses were conducted through the lens of Colombian healthcare and included only direct medical costs. Health outcomes were reported as quality-adjusted life-years (QALYs), and economic outcomes as incremental cost-effectiveness ratios (ICERs) and net monetary benefit (NMB). The study adhered to the Consolidated Health Economic Evaluation Reporting Standards (CHEERS 2022) [10].

### 2.2. Population and Clinical Context

The patient population consisted of adult immunocompromised patients with invasive aspergillosis (IA) or mucormycosis. These patients included individuals with hematologic malignancies, prolonged neutropenia, hematopoietic stem-cell transplantation, solid-organ transplantation, uncontrolled diabetes mellitus, and other underlying conditions associated with a high risk of invasive fungal disease. Given the important differences in epidemiology, disease progression, mortality, and treatment response between invasive aspergillosis and mucormycosis, both diseases were modeled separately within the same analytical framework.

The model did not use individual patient-level data from a Colombian clinical cohort or a single healthcare institution. Instead, a hypothetical population of adult immunocompromised patients was simulated using demographic and clinical characteristics reported in pivotal randomized controlled trials, observational cohorts, and international clinical practice guidelines. For invasive aspergillosis, the principal efficacy and safety inputs were derived from the SECURE trial (Maertens et al., 2016) [5], studies evaluating posaconazole-based therapies, and contemporary international guidelines. For mucormycosis, clinical parameters were informed by the VITAL study (Marty et al., 2016) [6], the ECMM Global Guideline for the Diagnosis and Management of Mucormycosis, and complementary observational evidence.

Clinical outcomes incorporated into the model included treatment response, treatment failure, all-cause mortality, treatment-related toxicity, treatment discontinuation, and switching to alternative antifungal therapies. These probabilities were extracted from published evidence and supplemented with pooled estimates or weighted averages when multiple high-quality sources were available. Whenever possible, parameter selection prioritized systematic reviews, meta-analyses, randomized controlled trials, and evidence supported by guidelines.

Although no comprehensive Colombian comparative studies evaluating all antifungal strategies were identified, the economic evaluation was conducted from the perspective of the Colombian healthcare system. Consequently, while effectiveness, mortality, and toxicity parameters were derived from international clinical evidence, all costs, resource utilization estimates, and economic outcomes were adapted using Colombian healthcare databases, national drug-pricing repositories, and official tariff schedules.

To minimize confounding and facilitate comparisons across treatment strategies, baseline demographic and clinical characteristics were assumed to be comparable within each disease-specific model. Therefore, the simulated population represents a representative cohort of immunocompromised patients commonly encountered in routine clinical practice rather than a specific Colombian institutional population.

To facilitate comparison between treatment strategies and minimize confounding, baseline demographic and clinical characteristics were assumed to be comparable across treatment arms within each disease-specific model. For invasive aspergillosis, all simulated patients were assumed to have similar distributions of age, underlying immunosuppressive conditions, disease severity, and baseline mortality risk regardless of the antifungal strategy evaluated. The same assumption was applied within the mucormycosis model. However, no assumption of equivalence was made between invasive aspergillosis and mucormycosis populations, which were modeled separately to account for their distinct epidemiological and clinical characteristics.

### 2.3. Interventions and Comparators

#### Antifungal Tactics Analyzed Were the Following

Treatment comparators were selected in accordance with international clinical practice guidelines, published clinical evidence, and availability within the Colombian healthcare system.

For invasive aspergillosis, voriconazole and isavuconazole were considered the principal first-line treatment strategies. Posaconazole and caspofungin were evaluated as alternative or salvage therapies for patients experiencing treatment failure, intolerance, contraindications, or clinically significant drug interactions.

For mucormycosis, liposomal amphotericin B was considered the standard first-line therapy. Isavuconazole and posaconazole were evaluated as alternative or salvage treatment strategies, particularly for patients with intolerance, contraindications, and renal toxicity associated with amphotericin-based therapy or as step-down therapy following initial disease control. Caspofungin was not included in the mucormycosis model because echinocandins have limited activity against Mucorales and are not recommended as monotherapy for invasive mucormycosis according to current international guidelines.

### 2.4. Model and Clinical Relevance

The model consisted of the following two parts:

1. Decision Tree (Early Phase). The first decision tree recorded early clinical outcomes as part of the acute phase of treatment, including clinical response, treatment failure, toxicity-related discontinuation, and early mortality. This format permitted the showing of a true picture of the high-risk early period post-diagnosis, with the majority of deaths and life-threatening adverse events occurring during this period.
2. Markov Model (Disease Progression). After the first phase, patients are followed using a Markov model with monthly cycles over a 6-month time horizon. The model contained four mutually exclusive health states: clinical response, treatment failure, toxicity/discontinuation (with switching or without switching), and death (absorbing state). The state transitions were determined by probabilities calculated from published clinical trials. The model was constructed by considering real-world clinical trajectories, particularly the roles of toxicological influences and treatment transfer in shaping outcomes.

### 2.5. Time Horizon and Cycle Length

A 6-month duration was chosen to capture the most clinically relevant outcomes, including early mortality, treatment response, toxicity, and resource utilization. The time period is characterized by initial induction and early consolidation of antifungal therapy, during which most clinical and economic interventions occur. Monthly cycles were used in the Markov model to balance computational and clinical feasibility. Discounting has not been employed for the short interval of the investigation.

### 2.6. Clinical Inputs

Clinical parameters were obtained through a targeted literature review of randomized controlled trials, observational studies, meta-analyses, and international clinical practice guidelines. For invasive aspergillosis, efficacy and safety data were primarily derived from the SECURE trial (Maertens et al., 2016) [5]. and the phase III trial comparing posaconazole with voriconazole (Maertens et al., 2021) [11]. For mucormycosis, clinical outcomes were informed by the VITAL study (Marty et al., 2016) [6] and the ECMM Global Guideline on Mucormycosis (Cornely et al., 2019) [3]. Mortality, treatment response, toxicity rates, and treatment-switching probabilities were extracted from these studies and supplemented with pooled estimates from observational cohorts when necessary. When multiple data sources were available, weighted averages were calculated based on study size and methodological quality.

Clinical response was defined as a composite outcome incorporating both clinical improvement and radiological stabilization or improvement, consistent with endpoint definitions used in pivotal antifungal clinical trials and international guideline recommendations.

Parameters were used based on the prioritization of various data sources that were available: pooled estimates from meta-analyses, large randomized controlled trials, guideline-referenced values, and well-designed observational studies. The main clinical outcomes were treatment response (6 to 12 weeks), all-cause mortality, severe toxicity (renal and hepatic toxicity), discontinuation, and switching of treatment. Pooled estimates or weighted averages were employed where appropriate. Parameter uncertainty was accounted for using confidence intervals or plausible ranges reported in the literature. Table 1A,B, presents the clinical inputs and parameter values used for invasive aspergillosis and mucormycosis (Table 1).

A complete description of all model parameters, evidence sources, probability distributions, and assumptions used in deterministic and probabilistic sensitivity analyses is provided in Supplementary Table S1.

Following reviewer feedback and in accordance with current ECMM and international guideline recommendations, caspofungin was excluded from the mucormycosis model because echinocandins have limited activity against Mucorales and are not recommended as monotherapy for invasive mucormycosis.

Clinical parameters were derived from pivotal randomized clinical trials (SECURE and VITAL), international clinical practice guidelines (IDSA and ECMM), and supplementary observational studies. When multiple sources were available, weighted averages were calculated according to sample size and methodological quality.

### 2.7. Costs

Direct medical costs included antifungal drug acquisitions, hospitalization (ward and intensive care), management of adverse events, monitoring, and follow-up.

Drug acquisition costs were obtained from the Colombian Drug Price Information System (SISMED) using the most recent complete dataset available (as of 2024). For each antifungal agent, the median institutional purchase price was used to minimize the influence of extreme values and reflect routine procurement practices within the Colombian healthcare system. Hospitalization costs, including general ward and intensive care unit (ICU) stays, were obtained from the SOAT 2025 tariff schedule and supplemented with adjusted ISS tariff references as needed. All costs were standardized to 2025 Colombian pesos (COP) using the Colombian Consumer Price Index (CPI). Resource utilization estimates were derived from published studies, clinical guidelines, and expert assumptions regarding clinical practice (Supplementary Table S2: Cost Inputs and Data Sources).

Detailed assumptions about healthcare resource utilization are presented in Supplementary Table S3. Resource consumption estimates included hospitalization days, intensive care unit stay, laboratory monitoring, specialist consultations, imaging studies, and management of treatment-related toxicities. These inputs were combined with Colombian unit costs to estimate total healthcare expenditures for each antifungal strategy.

### 2.8. Data on Institutional Procurement

In this work, all costs were expressed in 2024–2025 Colombian pesos (COP) and then converted to US dollars (USD) at a rate of COP 4100 per USD. Due to the short time horizon, costs were not discounted either.

### 2.9. Health Outcomes and Utilities

The health outcomes were assessed in quality-adjusted life-years (QALYs), defined as the total time spent in each health state, multiplied by its utility weight. Utility values were reflected in published studies of other patients with invasive fungal infections or similar diseases. States of toxicity or treatment failure were allocated lower utility values, indicating decreased quality of life.

### 2.10. Base-Case Analysis

Base-case analysis estimated total costs, QALYs, and ICERs for each antifungal strategy. Strategies were ordered for increasing cost and dominated or extensively dominated strategies were excluded. Stepwise incremental analysis was conducted to derive the best strategies for the cost-effectiveness frontiers.

### 2.11. Sensitivity Analyses

Both deterministic and probabilistic sensitivity analyses were performed to evaluate parameter uncertainty.

### 2.12. Deterministic Sensitivity Analysis

One-way sensitivity analyses were conducted by varying the main parameters across plausible values; tornado diagrams were used to summarize the results and identify the most influential variables.

### 2.13. Probabilistic Sensitivity Analysis (PSA)

A probabilistic sensitivity analysis was conducted using 10,000 Monte Carlo simulations. Probability distributions were assigned to model parameters as follows: beta distributions for probabilities and utilities, gamma distributions for costs, and log-normal distributions for relative effects.

The results were summarized using cost-effectiveness acceptability curves (CEACs), cost-effectiveness plans, and net monetary benefit analyses. Willingness to pay (WTP) thresholds of COP 44 million and COP 75 million per quality-adjusted life-year (QALY) gained were applied [18]. The lower threshold was based on health opportunity-cost estimates proposed for middle-income countries by Woods et al. [19] and Ochalek et al. [20]. In contrast, the higher threshold represents a benchmark commonly used in economic evaluations conducted in Latin American healthcare systems. These thresholds were selected to reflect alternative decision-making scenarios relevant to the Colombian healthcare context.

### 2.14. Net Monetary Benefit

Net monetary benefit (NMB) was calculated using the following formula:

NMB = (QALYs × WTP threshold) − Total cost

where QALYs represent quality-adjusted life-years, WTP represents the willingness-to-pay threshold per QALY gained, and total cost represents the expected direct medical cost of each antifungal strategy. A strategy with a higher NMB was considered economically preferred at a given WTP threshold. NMB was estimated for each antifungal strategy at WTP thresholds of COP 44 million and COP 75 million per QALY.

### 2.15. Scenario and Threshold Analyses

In addition to the following alternative assumptions, scenario analyses were performed to assess their impact: variations in drug pricing, shorter treatment durations, and estimates of real-world effectiveness. Threshold analyses were conducted to assess the highest drug price at which each strategy would be cost-effective at preset WTP thresholds.

### 2.16. Use of AI Tools

AI tools were used solely for language editing and manuscript refinement. These tools were not involved in data generation, model building, parameter selection, or results interpretation.

The short-term decision tree outlines the initial clinical management of invasive fungal infections and details the acute-phase outcomes (early clinical response, treatment failure, toxicity, and death). Patients who survive the initial phase enter the Markov model, reflecting the progression of disease over stages via multiple health states—clinical response, treatment failure, toxicity/discontinuation, and death. This hybrid arrangement represents the high early mortality and changing clinical course typical of invasive aspergillosis and mucormycosis.

**Figure 1:**
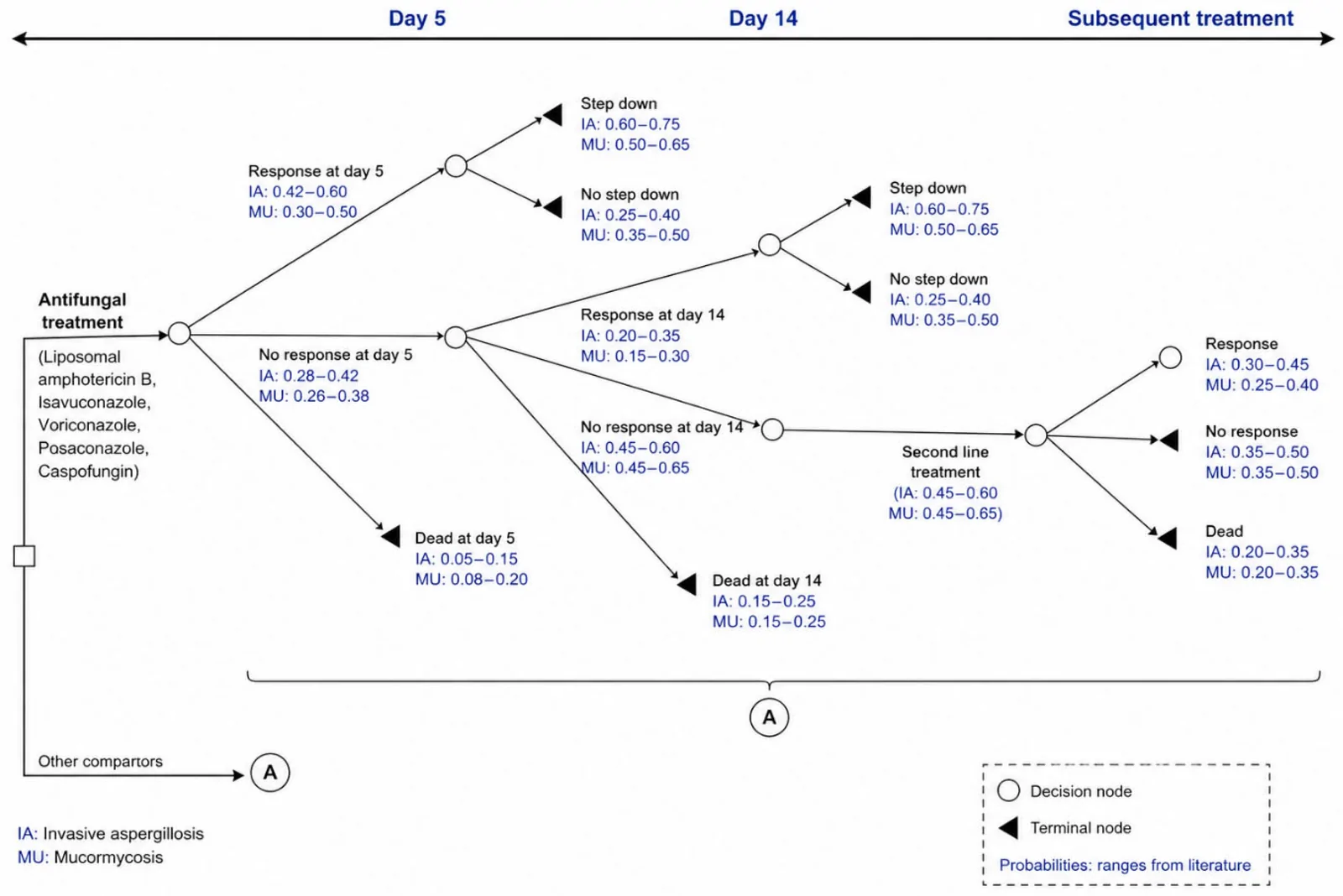
Hybrid decision tree and Markov model structure for antifungal treatment strategies in invasive fungal infections.

Step-down antifungal treatment was defined as the transition from initial intravenous antifungal therapy to oral antifungal therapy after clinical stabilization, evidence of treatment response, and absence of uncontrolled disease progression. In the model, step-down therapy represented continuation or consolidation therapy after the acute treatment phase. The structure of the hybrid decision tree and Markov model used in the analysis is presented in Figure 1.

## 3. Results

### 3.1. Outcomes in the Base Case: Clinical and Economic

We used base-case analysis to identify clinically and economically relevant differences between antifungal strategies in invasive aspergillosis and mucormycosis. From a clinical standpoint, the most significant differences were observed in early survival, treatment response, and toxicity-related transitions. In contrast, overall differences in long-term quality-adjusted survival were relatively small over the 6-month horizon. The base-case cost–utility results for invasive aspergillosis and mucormycosis are summarized in Table 2.

### 3.2. Invasive Aspergillosis

In invasive aspergillosis, triazole-administered therapies led to better overall clinical trajectories relative to amphotericin-based management. Voriconazole or isavuconazole was associated with significantly higher rates of patients remaining in the clinical response group over time, with lower rates of toxicity-related cessation and hospitalization. At 6 months, isavuconazole provided the largest health benefit (0.334 QALYs) in contrast to voriconazole (0.312 QALYs), at an incremental benefit of 0.022 QALYs (~8 additional days of perfect health). This benefit was largely attributed to enhanced tolerability and fewer transitions to halt due to toxicity rather than marked disparities in early mortality. Regarding cost, voriconazole costs COP 38.2 million (USD 9550), while isavuconazole costs COP 41.6 million (USD 10,400), representing an incremental cost of COP 3.4 million (USD 850). The accompanying incremental cost-effectiveness ratio (ICER) for isavuconazole versus voriconazole was COP 154.5 million per QALY (USD 38,636 per QALY). Liposomal amphotericin B and caspofungin were either dominated or extensively dominated due to higher costs and lower effectiveness. Posaconazole showed an intermediate performance but also dominated in the incremental analysis.

### 3.3. Mucormycosis

In mucormycosis, clinical features were markedly different, suggesting a more aggressive disease. Liposomal amphotericin B provided the maximum benefit of survival in the form of 0.315 QALYs, compared to 0.280 QALYs for isavuconazole and 0.285 QALYs for posaconazole. The incremental benefit of liposomal amphotericin B on isavuconazole was 0.035 QALYs (~12.8 days of perfect health), mostly due to decreased early mortality rates during the early cycles of treatment. However, this clinical benefit was offset by a massive increase in cost, with total spending of COP 61.8 million (USD 15,450) and COP 44.5 million (USD 11,125) for isavuconazole. They reported an ICER of COP 494.3 million per QALY (USD 123,571 per QALY), highlighting the substantial financial cost of incremental survival in rapidly progressive infection. Posaconazole showed a marginal incremental benefit versus isavuconazole and was poorly cost-effective.

#### Cost Composition and Resource Utilization

Acquired antifungal drugs and hospitalization costs in both disease categories accounted for the majority of total costs. Drug purchases represented 60–72% of total costs and were the largest in the liposomal amphotericin B–based regimens due to high unit costs and the need for intravenous drug delivery. Hospitalization was the second-largest cost component (15–25%), particularly in strategies that required prolonged intravenous therapy. In contrast, liposomal amphotericin B and caspofungin were associated with increased inpatient resource use, whereas triazole-based strategies enabled earlier transfer of care to outpatient management and reduced hospitalization costs. The costs of toxicity management represented about 5–10% of total expenses. Cumulative costs related to nephrotoxicity were more common with amphotericin-based regimens, and costs associated with hepatotoxicity and drug–drug interactions were more common with azole-based therapies. Monitoring and follow-up costs were relatively low (<10%) and contributed only marginally to incremental comparisons.

### 3.4. Markov Model Dynamics and Clinical Trajectories

The Markov model showed that clinically relevant transitions occurred most frequently within the first 2 cycles, underscoring the importance of early therapeutic efficacy in invasive fungal infections. However, in invasive aspergillosis, the azole regimen remained effective over time, with a higher proportion of patients in clinical response. At month 1, 58–62% of patients receiving either voriconazole or isavuconazole were still responding (compared to around 50% for liposomal amphotericin B), and between months 1 and 6, there were higher response rates for isavuconazole (42%) and voriconazole (39%) in comparison with the alternative agents. The 6-month cumulative mortality varied from 32 to 35% for triazole-based regimens to over 40% for amphotericin-based regimens. These differences, while clinically important, yielded modest QALY improvements over such a small analytic window.

In mucormycosis, the disease progression was more aggressive, with 30–40% of patients dying in the first cycle, depending on the strategy. Liposomal amphotericin B showed lower rates of early death (about 45% of patients survived and responded at month 1), compared with 35–38% response rates with azole-based alternatives. At month 6, the highest survival was observed with liposomal amphotericin B (30%) compared with isavuconazole and posaconazole (24–26%). Table 3 presents the clinical and economic outcomes generated by the Markov model over the 6-month time horizon (Table 3).

Outcomes were derived from the Markov model using monthly cycles over a 6-month horizon. “Alive in response” represents the proportion of patients remaining in the clinical response state at the end of the simulation. Cumulative mortality reflects all-cause mortality over the analytic period. The time to response corresponds to the mean duration spent in the clinical response state.

Duration of Clinical Response represents the average time spent in the clinical response health state within the Markov model following successful treatment. Clinical response was defined as a composite endpoint comprising clinical improvement and radiological stabilization or improvement, without evidence of treatment failure or disease progression.

### 3.5. Net Monetary Benefit and Decision Uncertainty

The net monetary benefit (NMB) analysis demonstrated that cost-effectiveness conclusions varied depending on the willingness-to-pay (WTP) threshold. In invasive aspergillosis, voriconazole achieved the highest NMB at conservative thresholds (COP 44 million/QALY), supporting its economic preference in resource-constrained settings. Isavuconazole became increasingly competitive at higher thresholds (COP 75 million/QALY), reflecting its improved tolerability and marginal QALY gains.

**Figure 2:**
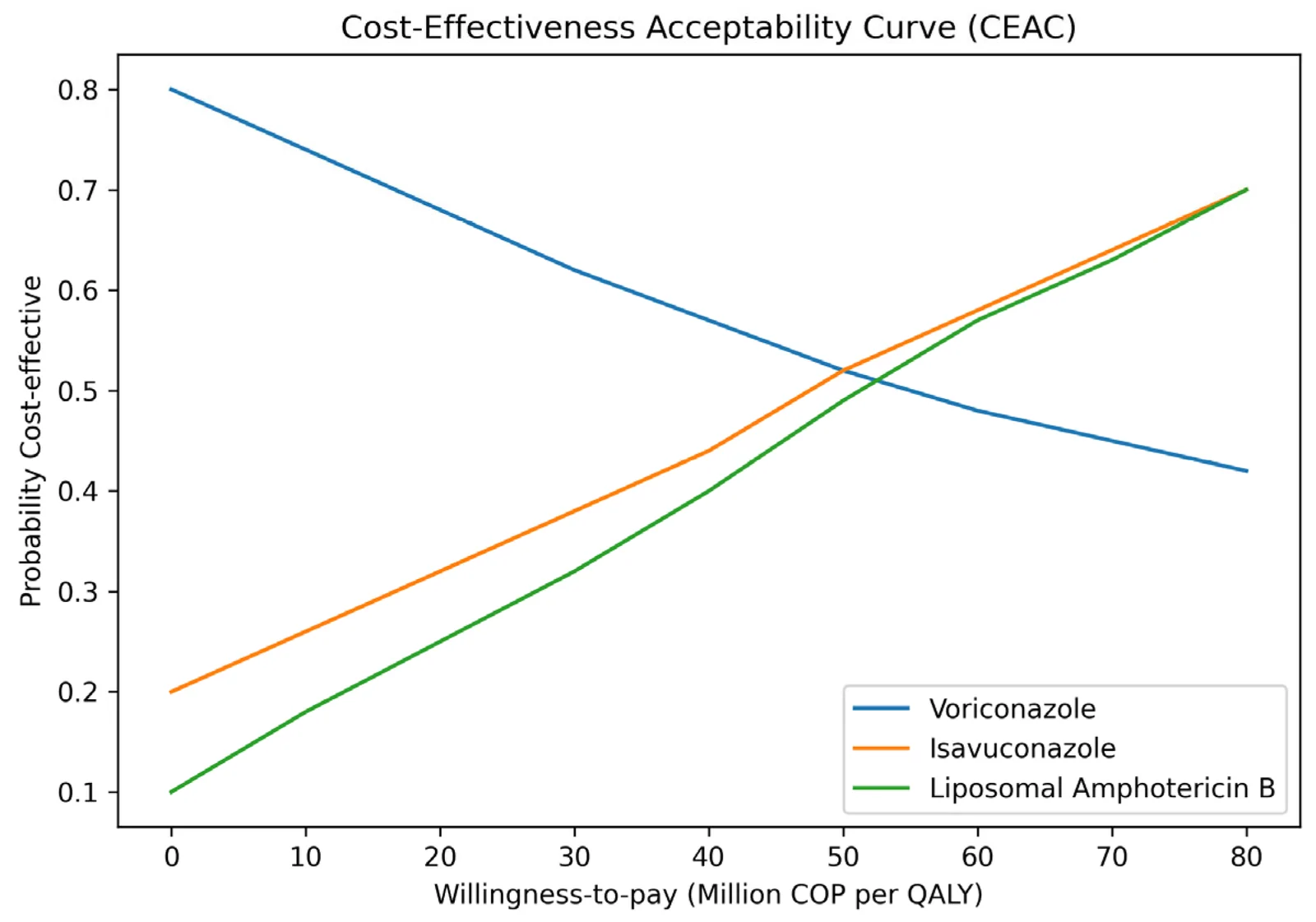
Cost-effectiveness acceptability curves (CEACs) for antifungal strategies in invasive aspergillosis and mucormycosis.

In mucormycosis, isavuconazole consistently yielded the highest NMB across a wide range of thresholds due to its lower cost profile. Liposomal amphotericin B became competitive only at higher WTP values, reflecting a trade-off between survival benefit and substantially higher cost. The cost-effectiveness acceptability curves for the evaluated antifungal strategies are presented in Figure 2.

The curves represent the probability that each antifungal strategy is cost-effective at increasing willingness-to-pay thresholds per quality-adjusted life-year (QALY) gained. Voriconazole shows a higher probability of cost-effectiveness at lower thresholds, while isavuconazole and liposomal amphotericin B become more competitive at higher thresholds, reflecting trade-offs between cost, toxicity, and survival benefits.

### 3.6. Sensitivity Analyses

**Figure 3:**
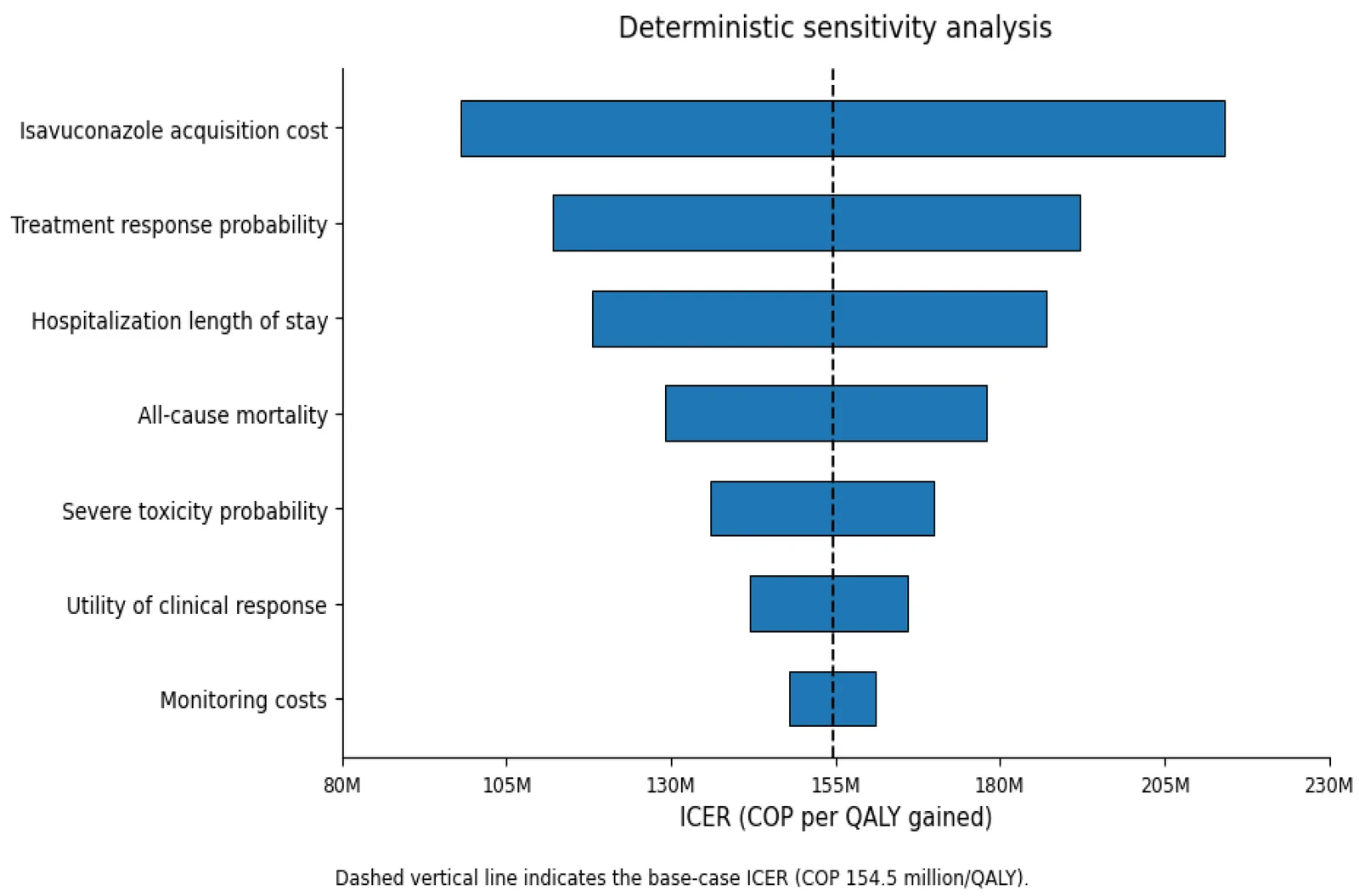
One-way sensitivity analysis (tornado diagram) for isavuconazole versus voriconazole.

Deterministic sensitivity analyses demonstrated that antifungal acquisition costs, treatment response probabilities, hospitalization duration, and all-cause mortality were the parameters with the greatest influence on incremental cost-effectiveness ratios (ICERs). As shown in Figure 3, variation in the acquisition cost of isavuconazole generated the widest range of ICER estimates, followed by treatment response rates and hospitalization length of stay. Mortality parameters had a moderate influence on model outcomes, whereas utility values and monitoring costs had comparatively smaller effects.

Probabilistic sensitivity analysis confirmed the robustness of the base-case findings. In invasive aspergillosis, voriconazole showed the highest probability of being cost-effective at lower willingness-to-pay (WTP) thresholds, whereas isavuconazole became increasingly favorable as WTP increased. In mucormycosis, isavuconazole demonstrated the highest probability of cost-effectiveness across most threshold values, although liposomal amphotericin B became competitive at higher WTP thresholds due to its survival advantage.

Scenario analyses further showed that reductions of approximately 20–25% in the acquisition cost of isavuconazole substantially improved its cost-effectiveness profile, reducing the ICER to levels closer to conventional Colombian willingness-to-pay thresholds.

The tornado diagram illustrates the effect of varying individual model parameters across predefined ranges while holding all other variables constant. The vertical reference line represents the base-case incremental cost-effectiveness ratio (ICER) of COP 154,545,455 per quality-adjusted life-year (QALY) gained. The widest bars indicate the parameters with the greatest influence on model outcomes. Antifungal acquisition costs, treatment response probabilities, hospitalization length of stay, and all-cause mortality were identified as the primary drivers of cost-effectiveness. Utility values and monitoring costs had a comparatively smaller impact on ICER estimates.

Tornado diagram showing the impact of varying individual model parameters on the ICER of isavuconazole versus voriconazole. The dashed vertical line represents the base-case ICER (COP 154.5 million/QALY).

## 4. Discussion

Herein, we report an integrative clinical and econometric assessment of antifungal strategies for invasive aspergillosis and mucormycosis underlined by the multidimensional context of early survival, toxicity burden, and health care resource consumption.

Our results show that cost-effectiveness in both settings is largely a function of early treatment events, not of long-duration differences in survival, which are constrained by the narrow context of a short analytic horizon—clinical interpretation and relevance. Isavuconazole, compared with voriconazole, demonstrated an incremental marginal benefit over invasive aspergillosis, the rationale not being a strong initial mortality difference but rather better tolerability and earlier non-remission. These results are consistent with the SECURE trial, which found that isavuconazole is non-inferior in mortality and has a more favorable side-effect profile and fewer adverse events [5].

In our model, this translated into a higher proportion of patients who did not regress during the disease course despite treatment, providing a relatively small but significant benefit for quality of service. In a clinical sense, the findings further support the notion that antifungal decisions in invasive aspergillosis are not based solely on whether an intervention is efficacious but are also influenced by its maintenance and toxicity-related interference. In practical use, such consideration may be important due to the hepatotoxicity and drug–drug interaction of voriconazole that could adversely affect treatment maintenance [10,18,19,20,21,22,23].

It appears, then, that the economic benefit of isavuconazole is primarily attributable to its tolerability advantage rather than to its superior antifungal properties. On the other hand, the clinical–economic trade-off was more apparent in mucormycosis. Liposomal amphotericin B showed the greatest survival benefit, confirming its well-established role as first-line treatment in worldwide guidelines [4,5]. This observation is consistent with prior observations and consensus recommendations that amphotericin-based regimens provide better early survival in cases of severe disease [20].

This improved survival, though, came at significantly more cost in the form of drug acquisition, hospitalization, and management of toxicity. While isavuconazole was associated with lower survival in these patients, it consistently had a more favorable economic profile due to lower toxicity and reduced resource use. These data are consistent with results from the VITAL study and subsequent analyses suggesting that isavuconazole may yield similar outcomes in selected patient populations, especially those unable to tolerate amphotericin-based therapy [7,8,9].

### 4.1. Comparison with Earlier Studies

Our results corroborate prior studies in high- and middle-income countries that found drug acquisition costs, hospitalization, and adverse event management to be leading contributors to total costs in invasive fungal infections [4,5,6]. The cost-effectiveness of isavuconazole compared to voriconazole has also been reported, adjusting for specific pricing and hospitalization, using studies from China and Brazil [7,8,9].

Crucially, our study contributes to this evidence by comparing several antifungal treatments within a single model framework for two different invasive fungal infections at a single site and combining these treatments with a single treatment mode; this serves to easily enable direct comparison of clinical-economic trade-offs across disease, clinical costs, and benefits among different conditions, reinforcing the need for treatment response to be based on disease severity and early mortality dynamics based on cost-effectiveness.

Although previous economic evaluations have compared selected antifungal therapies in high-income countries, evidence from Latin America remains limited. To our knowledge, this study represents the first Colombian cost-utility analysis to simultaneously evaluate multiple antifungal strategies for both invasive aspergillosis and mucormycosis, incorporating toxicity, hospitalization requirements, treatment switching, net monetary benefit, and healthcare resource utilization within a unified modeling framework. Consequently, the added value of this study lies not in confirming established efficacy findings but in quantifying the clinical–economic consequences of treatment selection under the constraints of a middle-income healthcare system [14,24,25,26,27].

### 4.2. Health System Implications

These results provide important findings that are relevant for clinical decision-making and health policy (especially in resource-constrained settings). The high early mortality and extensive initial costs of invasive fungal infections make treatment decisions most of the time a matter of cost–benefit analysis compared to a survivor’s ultimate survival. Strategies that limit hospitalizations and toxic effects, such as triazole treatment regimens, might be a less burdensome option in scenarios of limited medical resources, even if incremental survival benefits are small.

In contrast to this, in severe infections, including mucormycosis, where early mortality is very high, the preservation of life using amphotericin-based therapy may be clinically justified despite higher costs. Therefore, our results argue for a context-based antifungal therapy strategy in which clinical guidelines, patient-specific variables, toxicity risk, and the capacity of health systems to contextualize treatment choices inform treatment choices.

### 4.3. Individualized Antifungal Decision-Making in Resource-Constrained Settings

The present analysis highlights that antifungal treatment decisions should not rely exclusively on efficacy estimates but should also consider patient-specific clinical characteristics and healthcare system constraints. In invasive aspergillosis, where mortality differences between modern triazole therapies are relatively modest, treatment selection may be influenced by toxicity profiles, drug–drug interactions, comorbidities, and anticipated hospitalization requirements. For example, isavuconazole may be particularly attractive for patients receiving multiple concomitant medications, those at risk of hepatotoxicity, or situations where minimizing inpatient resource utilization is a priority.

Conversely, in mucormycosis, early survival remains the dominant clinical objective. Liposomal amphotericin B demonstrated the greatest survival benefit and should remain the preferred strategy for critically ill patients, patients with extensive disease, or patients with rapidly progressive infections. However, in patients with significant renal dysfunction, intolerance to amphotericin-based regimens, or in settings where prolonged hospitalization is difficult to sustain, isavuconazole may represent a clinically acceptable alternative with a more favorable economic profile.

These findings suggest that optimal antifungal management should be individualized based on disease severity, organ function, anticipated toxicity risk, treatment goals, and healthcare resource availability. Rather than supporting a single universal strategy, the model provides a framework for balancing clinical benefit and economic sustainability in different healthcare scenarios.

### 4.4. The Performance of Uncertainty and Sensitivity Analyses

Sensitivity analyses confirmed that drug acquisition costs, treatment response rates, and hospitalization duration had the greatest influence on cost-effectiveness. These results highlight the importance of pricing strategies and their practical applications in assessing the value-creation effectiveness of an antifungal treatment. Notably, even modest cost reductions in isavuconazole were found to enhance its cost-effectiveness profile; therefore, price negotiation and institutional procurement strategies may be of importance in decision-making about therapy. These results confirm previous studies showing that the cost-effectiveness of antifungal drugs is sensitive to drug prices and healthcare systems [7,8,9,27,28,29,30].

An additional limitation is the absence of prospective clinical validation using real-world Colombian patient cohorts. Although the model was constructed using high-quality evidence from randomized controlled trials, observational studies, and international clinical guidelines [1,4,5,6], future studies should evaluate the external validity of these findings in multicenter healthcare settings. Validation using institutional databases and real-world clinical outcomes would refine model assumptions and improve the applicability of the results to routine clinical practice within the Colombian healthcare system.

### 4.5. Limitations and Sources of Bias

There are numerous caveats to interpreting these findings. The 6-month time horizon, while appropriate in accounting for early mortality and treatment-induced toxicity, may underestimate long-term benefits associated with sustained disease control. The major clinical events have to arise at this time due to the high early mortality resulting from invasive fungal infections.

Second, clinical input parameters were obtained from selected randomized trials, observational studies, and guideline-based estimates. While this model captures the availability of real-world evidence, it introduces heterogeneity and potential biases across patient populations, disease severity, and treatment modalities.

Third, utility values were derived from published studies of invasive fungal infections and similar diseases, which may not fully capture the quality-of-life effects of these diseases. This limitation is typical in economic evaluations of rare or very severe infections and may influence absolute QALY estimates.

Fourth, the cost estimates were conducted using Colombian data and may not be directly generalizable to other healthcare systems. Nevertheless, the differences between strategies in these cases are likely to be held similar between settings with similar resource limitations. Last but not least, structural assumptions, including the definition of health states and the omission of long-term suppressive therapy from the model, were likely relevant to the results.

While probabilistic sensitivity analyses were conducted to address parameter uncertainty, intrinsic structural uncertainty persists in decision-analytic models. Conclusion. Conclusions: This study underscores the importance of early survival, toxicity burden, and resource utilization in assessing the clinical and economic value of antifungal therapy for invasive aspergillosis and mucormycosis.

Although isavuconazole offers moderate clinical benefits and improved tolerability in invasive aspergillosis, liposomal amphotericin B remains the most efficacious option for mucormycosis, albeit at a substantially higher cost. The results suggest that individualized, situation-specific treatment decision-making based on clinical effectiveness, toxicity risk, and healthcare resource limitations should take precedence. This study thereby provides the necessary evidence linking the clinical and economic perspectives to support the rational choice of antifungal therapy in clinical practice and health policy.

### 4.6. Future Research Directions

Future research should focus on validating the present model using multicenter real-world data from Colombian hospitals and healthcare institutions. Such studies could evaluate the concordance between modeled and observed clinical outcomes, including mortality, treatment response, toxicity, hospital length of stay, and healthcare costs. In addition, prospective registries of invasive fungal infections would facilitate the development of locally calibrated economic models and support more individualized antifungal treatment strategies.

### 4.7. Implications for Clinical Practice and Decision-Making in Policy Formulation

Considering these limitations, this review demonstrates that the economic-clinical trade-offs inherent to antifungal therapy are transparently quantified. For invasive aspergillosis, where efficacy differences are small, economic value is derived from avoiding toxicity-related risks and lower hospitalization costs. Compared with amphotericin-based therapies, the economic significance of survival benefits in mucormycosis underscores the need for greater efficiency of isavuconazole therapy, particularly for specific patient groups or in resource-limited settings. These results suggest a need for context-dependent therapeutic choices rather than a one-size-fits-all approach and underscore the importance of matching antifungal therapy to clinical severity and the health care delivery system’s capacity.

## Figures and Tables

**Table 1:** (**A**) Clinical inputs for invasive aspergillosis. (**B**) Clinical inputs for mucormycosis.

(A)			
Parameter	Base Value	Range Used in Sensitivity Analysis	Source
Treatment response probability	0.61	0.55–0.67	SECURE Trial [5]
All-cause mortality	0.29	0.20–0.35	SECURE Trial [5]
Severe toxicity probability	0.18	0.10–0.25	Maertens et al. [5]; Cornely et al. [7]
Treatment discontinuation due to adverse events	0.12	0.08–0.18	SECURE Trial [5]; Cornely et al. [7]
Treatment-switch probability	0.18	0.10–0.25	Andes et al. [8]; Lewis and Kontoyiannis [12]
Utility—Clinical response state	0.82	0.70–0.85	Harrington et al. [12]; Gold et al. [9]; Neumann et al. [13]
Utility—Progressive disease state	0.52	0.40–0.65	Harrington et al. [12]; Gold et al. [9]; Neumann et al. [13]
Utility—Severe toxicity state	0.45	0.30–0.60	Harrington et al. [12]; Gold et al. [9]
Durability of clinical response (months)	18	12–24	SECURE Trial [5]; Posaconazole Trial [14]; Andes et al. [8]
Hospitalization duration (days)	18	12–24	Andes et al. [8]; Neofytos et al. [15]
ICU stay (days)	4	2–8	Neofytos et al. [15]; Pagano et al. [16]
**(B)**			
**Parameter**	**Base Value**	**Range Used in Sensitivity Analysis**	**Source**
Treatment response probability	0.58	0.50–0.66	VITAL Trial [6]
All-cause mortality	0.43	0.35–0.55	VITAL Trial [6]; ECMM Guideline [3]
Severe toxicity probability	0.22	0.15–0.30	Spellberg et al. [17]; Cornely et al. [3]
Treatment discontinuation due to adverse events	0.15	0.10–0.22	Spellberg et al. [17]; Marty et al. [6]
Treatment-switch probability	0.12	0.05–0.20	Marty et al. [6]; Cornely et al. [3]
Utility—Clinical response state	0.82	0.70–0.85	Harrington et al. [12]; Gold et al. [9]; Neumann et al. [13]
Utility—Progressive disease state	0.52	0.40–0.65	Harrington et al. [12]; Gold et al. [9]; Neumann et al. [13]
Utility—Severe toxicity state	0.45	0.30–0.60	Harrington et al. [12]; Gold et al. [9]
Durability of clinical response (months)	14	8–20	VITAL Trial [6]; Spellberg et al. [17]
Hospitalization duration (days)	24	18–35	Marty et al. [6]; Spellberg et al. [17]
ICU stay (days)	8	4–12	Marty et al. [6]; Cornely et al. [3]

Abbreviations: ECMM, European Confederation of Medical Mycology; SECURE, phase III trial comparing isavuconazole and voriconazole; VITAL, phase III study evaluating isavuconazole in mucormycosis. Caspofungin was evaluated only in invasive aspergillosis as salvage therapy. It was not included in the mucormycosis model because current international guidelines do not support its use as monotherapy against Mucorales.

**Table 2:** Base-case cost–utility results of antifungal strategies for invasive aspergillosis and mucormycosis over a 6-month time horizon.

(A) Invasive Aspergillosis								
Strategy	Total Cost (COP)	Total Cost (USD)	QALYs	Incremental Cost (COP)	Incremental QALYs	ICER (COP/QALY)	ICER (USD/QALY)	Interpretation
Voriconazole	38,200,000	9550	0.312	—	—	—	—	Reference
Isavuconazole	41,600,000	10,400	0.334	3,400,000	0.022	154,545,455	38,636	Cost-effective at higher WTP
Posaconazole	45,900,000	11,475	0.320	Dominated	—	—	—	Dominated
Caspofungin	48,300,000	12,075	0.298	Dominated	—	—	—	Dominated
Liposomal Amphotericin B	62,700,000	15,675	0.305	Extendedly dominated	—	—	—	Excluded
**(B) Mucormycosis**								
**Strategy**	**Total Cost (COP)**	**Total Cost (USD)**	**QALYs**	**Incremental Cost (COP)**	**Incremental QALYs**	**ICER (COP/QALY)**	**ICER (USD/QALY)**	**Interpretation**
Isavuconazole	44,500,000	11,125	0.280	—	—	—	—	Reference
Posaconazole	46,200,000	11,550	0.285	1,700,000	0.005	340,000,000	85,00	Not cost-effective
Liposomal Amphotericin B	61,800,000	15,450	0.315	17,300,000	0.035	494,285,714	123,571	Conditionally cost-effective

ICER: Incremental cost-effectiveness ratio; QALYs: Quality-adjusted life-years. Costs are expressed in 2024–2025 Colombian pesos (COP) and converted to US dollars (USD) at a rate of COP 4100 per USD. “Dominated” indicates higher cost and lower effectiveness. “Extended dominance” indicates a strategy that is less efficient than a combination of other alternatives. Cost-effectiveness interpretation is based on willingness-to-pay (WTP) thresholds of COP 44 million and COP 75 million per QALY.

**Table 3:** Markov model outcomes by antifungal strategy for invasive aspergillosis and mucormycosis over a 6-month time horizon.

(A) Invasive Aspergillosis						
Strategy	Alive in Response at Month 6 (%)	Cumulative Mortality at Month 6 (%)	Duration of Clinical Response (Months)	QALYs (6 Months)	Total Cost (COP)	Total Cost (USD)
Voriconazole	39%	34%	3.8	0.312	38,200,000	9550
Isavuconazole	42%	32%	4.1	0.334	41,600,000	10,400
Posaconazole	36%	37%	3.6	0.320	45,900,000	11,475
Caspofungin	33%	40%	3.3	0.298	48,300,000	12,075
Liposomal Amphotericin B	35%	42%	3.5	0.305	62,700,000	15,675
**(B) Mucormycosis**						
**Strategy**	**Alive in Response at Month 6 (%)**	**Cumulative Mortality at Month 6 (%)**	**Duration of Clinical Response (Months)**	**QALYs (6 Months)**	**Total Cost (COP)**	**Total Cost (USD)**
Isavuconazole	25%	55%	2.9	0.280	44,500,000	11,125
Posaconazole	26%	53%	3.0	0.285	46,200,000	11,550
Liposomal Amphotericin B	30%	48%	3.4	0.315	61,800,000	15,450

## Data Availability

All data used in this study were obtained from publicly available sources, including published literature, national epidemiological reports, and official pricing repositories. Drug pricing information was obtained from the Ministry of Health of Colombia https://www.minsalud.gov.co/Paginas/default.aspx (accessed on 3 February 2026). All datasets supporting the findings of this study are included within the manuscript.

## Supplementary Materials

The following supporting information can be downloaded at https://www.mdpi.com/article/10.3390/jof12080608/s1: Figure S1. Detailed decision tree structure for invasive aspergillosis and mucormycosis; Table S1. Model input parameters and ranges used in sensitivity analyses. Table S2. Cost Inputs and Data Sources Used in the Economic Evaluation. Table S3. Healthcare Resource Utilization and Modeling Assumptions.

## Abbreviations

IA	Invasive aspergillosis
IM	Invasive mucormycosis
IFI	Invasive fungal infection
QALY	Quality-adjusted life year
ICER	Incremental cost-effectiveness ratio
WTP	Willingness-to-pay
COP	Colombian pesos
USD	United States dollars
TRM	Tasa Representativa del Mercado (Colombian market exchange rate)
PSA	Probabilistic sensitivity analysis
OWSA	One-way sensitivity analysis
CEAC	Cost-effectiveness acceptability curve
AEs	Adverse events
RCT	Randomized controlled trial
LMIC	Low- and middle-income countries
