# Supplementary material for: Clinical and Economic Trade-Offs in Antifungal Therapy for Invasive Aspergillosis and Mucormycosis: A Markov Model–Based Analysis
Source: J Fungi (Basel). 2026 Aug 14;12(8):608. doi: 10.3390/jof12080608 (PMC13514336; doi:10.3390/jof12080608)
Supplement: Supplementary file 1 [file jof-12-00608-s001.zip › jof-4320330-supplementary.pdf]

Supplementary Table S1. Model input parameters used in the hybrid decision tree–Markov economic evaluation

Parameter	Base-case value	Range used in sensitivity analysis	Probability distribution	Data source
**Clinical parameters**				
Clinical response probability – Voriconazole	Trial estimate	±20%	Beta	Maertens et al., 2016
Clinical response probability – Isavuconazole	Trial estimate	±20%	Beta	Maertens et al., 2016
Clinical response probability – Liposomal amphotericin B	Published estimate	±20%	Beta	Cornely et al., 2019
Clinical response probability – Posaconazole	Published estimate	±20%	Beta	Cornely et al., 2019
Clinical response probability – Caspofungin	Published estimate	±20%	Beta	Patterson et al., 2016
Early mortality probability	Disease-specific	±20%	Beta	Clinical trials and guidelines
Treatment failure probability	Disease-specific	±20%	Beta	Literature synthesis
Probability of treatment discontinuation due to toxicity	Strategy-specific	±25%	Beta	Clinical trials
Probability of nephrotoxicity	Strategy-specific	±25%	Beta	Published studies
Probability of hepatotoxicity	Strategy-specific	±25%	Beta	Published studies
Probability of severe adverse event requiring hospitalization	Strategy-specific	±25%	Beta	Literature
**Utility parameters**				
Utility – Clinical response	Literature estimate	±10%	Beta	Published utility studies

Parameter	Base-case value	Range used in sensitivity analysis	Probability distribution	Data source
Utility – Treatment failure	Literature estimate	±10%	Beta	Published utility studies
Utility – Toxicity state	Literature estimate	±10%	Beta	Published utility studies
Utility decrement due to severe toxicity	Literature estimate	±10%	Beta	Literature
**Cost parameters (COP)**				
Voriconazole acquisition cost	Institutional price	±25%	Gamma	Colombian drug prices
Isavuconazole acquisition cost	Institutional price	±25%	Gamma	Colombian drug prices
Liposomal amphotericin B acquisition cost	Institutional price	±25%	Gamma	Colombian drug prices
Posaconazole acquisition cost	Institutional price	±25%	Gamma	Colombian drug prices
Caspofungin acquisition cost	Institutional price	±25%	Gamma	Colombian drug prices
Daily hospitalization cost	Institutional estimate	±25%	Gamma	Colombian hospital tariffs
Intensive care cost	Institutional estimate	±25%	Gamma	Colombian hospital tariffs
Laboratory monitoring cost	Institutional estimate	±20%	Gamma	Institutional data
Imaging cost	Institutional estimate	±20%	Gamma	Institutional data
Toxicity management cost	Institutional estimate	±25%	Gamma	Institutional data
**Model assumptions**				
Markov cycle length	1 month	Fixed	—	Model assumption
Time horizon	6 months	Scenario analyses (3–12 months)	—	Study design
Discount rate	3% annually	0–5%	Deterministic	International recommendations

Parameter	Base-case value	Range used in sensitivity analysis	Probability distribution	Data source
Number of Monte Carlo simulations	10,000	Fixed	—	Study design
Perspective	Healthcare system	Fixed	—	Study design

Abbreviations: COP, Colombian pesos; ICU, intensive care unit.

Notes: Transition probabilities were parameterized using beta distributions because probabilities are bounded between 0 and 1. Cost parameters were modeled using gamma distributions to reflect their right-skewed nature, whereas health-state utility values were modeled using beta distributions. Parameter ranges for deterministic sensitivity analyses were defined according to reported 95% confidence intervals whenever available or by applying clinically plausible variation (±20–25%) in the absence of published uncertainty estimates. All costs were expressed in 2025 Colombian pesos and converted to US dollars using the average annual exchange rate reported in the Methods section.

Figure S1. Detailed hybrid decision tree–Markov model structure for invasive aspergillosis and mucormycosis

The detailed structure of the hybrid decision tree–Markov model used to simulate the clinical pathways of patients with invasive aspergillosis and mucormycosis is presented in Supplementary Figure S1. The decision tree captures early treatment outcomes during the initial cycle, whereas the subsequent Markov component represents monthly transitions among clinical response, treatment failure, toxicity, and death over the 6-month analytic horizon.

During the first model cycle, patients may achieve a clinical response, experience treatment failure, discontinue treatment because of clinically significant toxicity, or die. Patients who survive the initial phase enter the Markov model in the health state corresponding to their early clinical outcome. During subsequent monthly cycles, patients may remain in the same non-death state, transition among clinical response, treatment failure, and toxicity, or move to death. Death is modeled as an absorbing state.

The Markov model uses a 1-month cycle length and a 6-month analytic horizon. Clinical response represents effective disease control during active antifungal therapy. Treatment failure represents persistent or progressive invasive fungal disease. Toxicity includes clinically significant antifungal-related adverse events, particularly nephrotoxicity, hepatotoxicity, electrolyte abnormalities, and treatment-limiting drug interactions. The combination-therapy branch for mucormycosis represents liposomal amphotericin B administered with posaconazole or another azole.

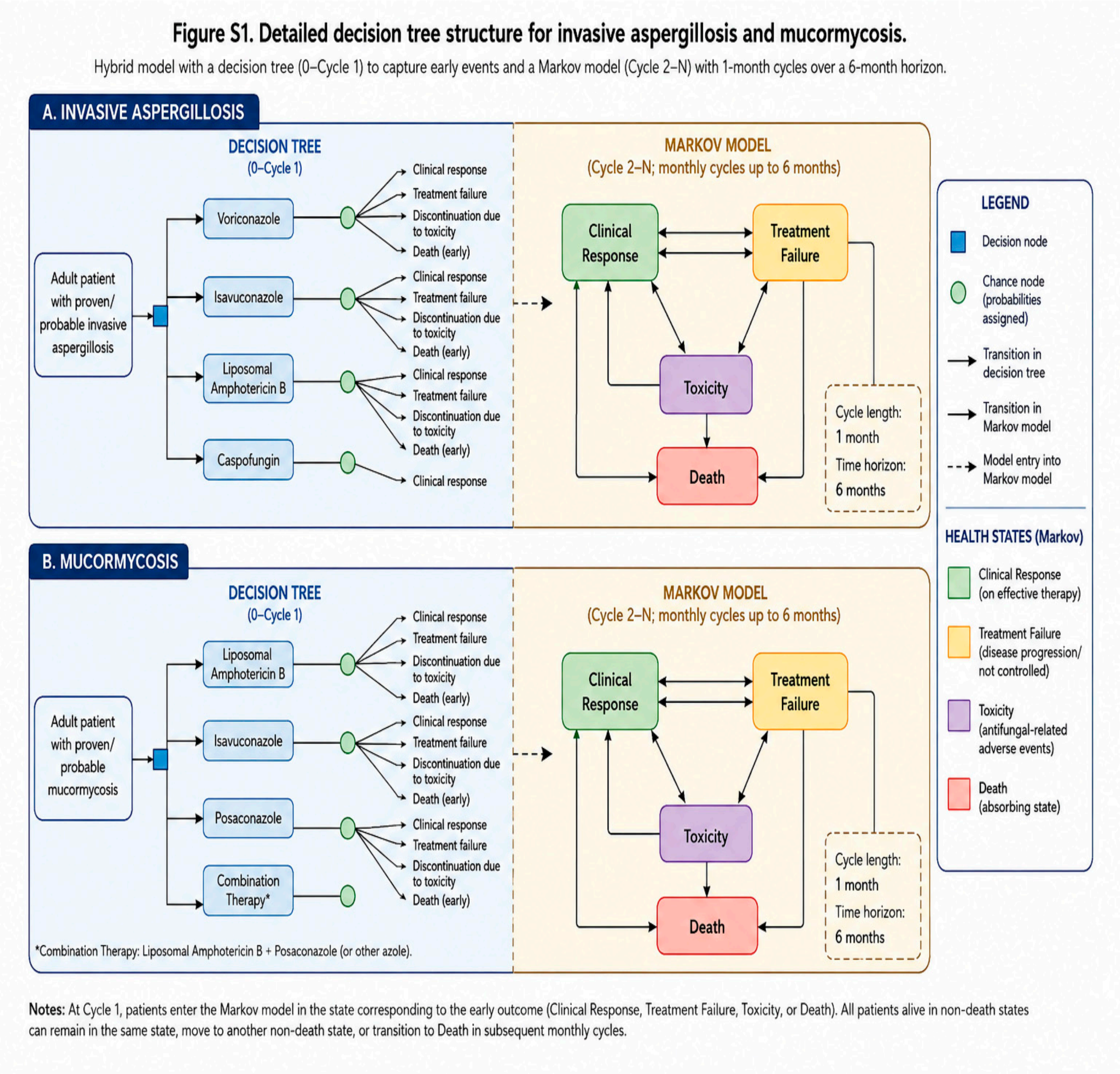

Figure S1. Detailed decision tree structure for invasive aspergillosis and mucormycosis.

Hybrid model with a decision tree (0–Cycle 1) to capture early events and a Markov model (Cycle 2–N) with 1-month cycles over a 6-month horizon.

Supplementary Figure S1 illustrates the hybrid decision tree–Markov model developed separately for invasive aspergillosis and mucormycosis. In the initial decision-tree phase, adult patients with proven or probable invasive fungal disease are assigned to one of the evaluated antifungal strategies. For invasive aspergillosis, the treatment alternatives include voriconazole, isavuconazole, liposomal amphotericin B, and caspofungin. For mucormycosis, the evaluated strategies include liposomal amphotericin B, isavuconazole, posaconazole, and combination therapy.

Blue squares indicate decision nodes at which the antifungal strategy is selected. Green circles represent chance nodes governed by treatment-specific probabilities. Solid arrows indicate transitions within the decision tree or Markov model, whereas dashed arrows indicate entry from the initial decision tree into the Markov component. Green boxes represent clinical response, yellow boxes represent treatment failure, purple boxes represent treatment-related toxicity, and red boxes represent death.

Transition probabilities, utility weights, and state-specific costs were assigned separately for each antifungal strategy according to clinical evidence and Colombian cost inputs. All patients entered the model at treatment initiation, and no transitions were allowed out of the death state.

Supplementary Table S2. Cost Inputs and Data Sources Used in the Economic Evaluation

Cost category	Resource / antifungal	Base-case input	Unit / duration	Source used in the model	Sensitivity range
**Antifungal acquisition**	Liposomal amphotericin B	USD 250/day	14-day initial IV induction phase	Colombian institutional procurement databases; SISMED cross-check	±25%
	Isavuconazole	USD 180/day	21-day initial modeled phase	Colombian institutional procurement databases; SISMED cross-check	±25%
	Voriconazole	USD 120/day	21-day initial modeled phase	Colombian institutional procurement databases; SISMED cross-check	±25%
	Posaconazole	USD 200/day	21-day initial modeled phase	Colombian institutional procurement databases; SISMED cross-check	±25%
	Caspofungin	USD 300/day	14-day initial IV phase	Colombian institutional procurement databases; SISMED cross-check	±25%
**Initial drug-course cost**	Liposomal amphotericin B	USD 3,500	Initial modeled induction	Derived from unit acquisition cost × modeled initial treatment duration	±25%
	Isavuconazole	USD 3,780	Initial modeled treatment phase	Derived from unit acquisition cost × modeled initial treatment duration	±25%

Cost category	Resource / antifungal	Base-case input	Unit / duration	Source used in the model	Sensitivity range
	Voriconazole	USD 2,520	Initial modeled treatment phase	Derived from unit acquisition cost × modeled initial treatment duration	±25%
	Posaconazole	USD 4,200	Initial modeled treatment phase	Derived from unit acquisition cost × modeled initial treatment duration	±25%
	Caspofungin	USD 4,200	Initial modeled induction	Derived from unit acquisition cost × modeled initial treatment duration	±25%
**General ward hospitalization**	Inpatient day	Model-specific Colombian tariff	Per day	ISS/SOAT tariff manuals and institutional tariff references	±20–25%
**Intensive care**	ICU day	Model-specific Colombian tariff	Per day	ISS/SOAT tariff manuals and institutional tariff references	±20–25%
**Renal toxicity management**	Laboratory monitoring, electrolyte replacement, nephrology care, dialysis where required	Event-specific model input	Per toxicity episode	Colombian national tariffs and published resource-use estimates	±20–25%
**Hepatic toxicity management**	Liver chemistry monitoring, specialist review, treatment modification	Event-specific model input	Per toxicity episode	Colombian national tariffs and published resource-use estimates	±20–25%
**Laboratory monitoring**	Renal, hepatic, electrolyte	Model-specific input	Per monitoring episode	ISS/SOAT and institutional tariffs	±20%

Cost category	Resource / antifungal	Base-case input	Unit / duration	Source used in the model	Sensitivity range
	and hematological laboratory tests				
**Diagnostic imaging**	CT and other indicated imaging	Model-specific input	Per procedure	Colombian national tariffs	±20%
**Specialist consultation**	Infectious diseases and other specialist assessment	Model-specific input	Per consultation	Colombian national tariffs	±20%
**Treatment switch**	Alternative antifungal following toxicity or failure	Drug-specific	Per switch episode	Drug acquisition cost plus associated monitoring/resource use	±25%
**Currency conversion**	COP to USD	1 USD = COP 4,100*	Applied to international reporting	Representative Colombian market exchange rate used in the original model	Fixed in base case

Supplementary Table S3. Healthcare Resource Utilization and Modeling Assumptions

Clinical/resource component	Base-case assumption	Model application	Main cost consequence	Sensitivity / scenario analysis
**Analytic horizon**	6 months	Applied to both invasive aspergillosis and mucormycosis	Captures early treatment, toxicity, mortality and subsequent care	3–12 months in structural scenarios
**Markov cycle length**	1 month	State transitions assessed monthly	Determines timing of cost and QALY accrual	Fixed
**Initial antifungal treatment**	All patients initiate assigned antifungal strategy	Initial treatment state	Drug acquisition + initial monitoring/hospitalization	Drug prices ±25%
**Clinical response**	Drug-specific response probabilities	Transition from treatment to response	Continued antifungal therapy and follow-up	Probability varied according to 95% CI or ±20%
**Treatment failure**	Drug-specific / pooled failure probability	Transition to failure state	Additional hospitalization and salvage therapy	±20%
**Clinically significant toxicity**	Renal or hepatic toxicity according to treatment	Transition to toxicity state	Additional monitoring, hospitalization and possible discontinuation	±20–25%
**Treatment switch following toxicity**	Approximately 70% in original transition framework	Toxicity → switch therapy	Alternative drug acquisition + monitoring	Varied in scenario analyses
**Death after severe toxicity**	Approximately 30% in original transition framework	Toxicity → death	Terminates future treatment expenditure and QALY accrual	Mortality varied ±15–20%
**Mortality following treatment failure**	Approximately 60% in original transition framework	Failure → death	Major determinant of QALY loss	Mortality varied ±15–20%
**Response after switch therapy**	Approximately 40%	Switch → response	Additional drug and follow-up costs with subsequent QALY accrual	±20%
**Hospitalization**	Required according to disease	Cost attached to initial treatment, toxicity and failure states	General ward cost	Length/cost varied ±20–25%

Clinical/resource component	Base-case assumption	Model application	Main cost consequence	Sensitivity / scenario analysis
	severity, IV administration and toxicity			
**ICU utilization**	Applied to patients with severe clinical deterioration or major toxicity	Severe failure/toxicity pathway	ICU cost per day	±20–25%
**IV antifungal administration**	Particularly relevant to L-AmB and caspofungin	Initial active treatment phase	Infusion and inpatient resource utilization	Duration varied in scenarios
**Oral step-down therapy**	Permitted when clinically appropriate	Response/switch pathway	Reduces prolonged inpatient administration costs	Alternative duration scenarios
**Laboratory monitoring**	Renal, hepatic, electrolyte and hematologic monitoring	Active treatment and toxicity states	Laboratory cost	±20%
**Imaging follow-up**	Periodic radiologic assessment	Response/failure monitoring	Imaging cost	±20%
**Specialist consultation**	Infectious diseases evaluation during active management	Initial, failure and toxicity states	Consultation cost	±20%
**Nephrotoxicity management**	Predominantly associated with L-AmB	Toxicity pathway	Monitoring, electrolyte replacement, possible dialysis	Toxicity probability and management cost ±20–25%
**Hepatotoxicity management**	Predominantly associated with azoles	Toxicity pathway	Laboratory follow-up and potential treatment change	Toxicity probability and management cost ±20–25%
**Drug–drug interactions**	Strategy-specific, particularly for azoles	May lead to treatment modification/discontinuation	Monitoring and switch costs	Strategy-specific range

Clinical/resource component	Base-case assumption	Model application	Main cost consequence	Sensitivity / scenario analysis
**Longer antifungal treatment**	Clinical therapy may extend ≥6–12 weeks	Captured within model horizon rather than interpreted as 14–21-day total therapy	Additional maintenance treatment and follow-up costs	Explicit duration scenarios recommended
**Discounting**	None in base case	6-month horizon	No material temporal adjustment	Not required because horizon <1 year
